# Effects of Short-Term Ageing Temperature on Conventional and High-Temperature Properties of Paving-Grade Bitumen with Anti-Stripping and WMA Additives

**DOI:** 10.3390/ma14216229

**Published:** 2021-10-20

**Authors:** Krzysztof Maciejewski, Piotr Ramiączek, Eva Remisova

**Affiliations:** 1 Department of Transportation Engineering, Faculty of Civil Engineering and Architecture, Kielce University of Technology, Al. Tysiąclecia Państwa Polskiego 7, 25-314 Kielce, Poland; piotrr@tu.kielce.pl; 2 Department of Highway and Environmental Engineering, Faculty of Civil Engineering, University of Zilina, Univerzitna 8215/1, 01001 Zilina, Slovakia; eva.remisova@fstav.uniza.sk

**Keywords:** bitumen, asphalt binder, ageing, RTFOT, short-term, anti-stripping, wax, WMA, additives, performance grade

## Abstract

The presented study explores the effects of decreased temperatures utilized in rolling thin-film oven (RTFOT) laboratory short-term ageing of asphalt binders based on 35/50- and 50/70-penetration paving-grade bitumen. Additionally, the effects of three additives used with these binders at different concentrations are evaluated: liquid anti-stripping agent, liquid warm-mix additive, and solid warm-mix additive. The resulting asphalt binders were subjected to basic (penetration at 25 °C, softening point, dynamic viscosity) and functional high-temperature characterization (G*/sin(δ), high critical temperature, non-recoverable creep compliance). It was found that the decreased short-term ageing temperatures may detrimentally impact the high-temperature grade of bituminous binders, but this effect can be mitigated by the use of appropriate additives. What is more, it was found that bituminous binders may respond differently to the aforementioned factors. Based on the results, it is advised that asphalt binders intended for use in warm-mix asphalts should be thoroughly tested to appropriately simulate the mixture production process and its effects.

## 1. Introduction

The road-construction industry nowadays faces expectations for decreasing the energy intensity of its operations while at the same time preserving or even increasing the quality and longevity of the produced infrastructure. These challenges arise not only from externally imposed regulations encouraging the shift towards a “green” economy but also by economic benefits and incentives of low-emission technologies. These conditions require the introduction of new, energy-efficient techniques incorporating new types of materials, such as reclaimed materials [1,2,3,4,5,6,7], reinforcement [8,9,10,11], and producing asphalt mixtures at decreased temperatures [12,13].

Warm-mix asphalt is typically produced at temperatures 20 °C to 30 °C lower than comparable hot mixtures [14], and it is expected to have properties no worse than those produced conventionally. In order to effectively coat the mineral mix at lower temperatures and to ensure proper workability and compactability of the resulting asphalt mixture, modifications in the production process are required. These alterations may include asphalt binder foaming [15,16], asphalt binder additives and binder fluxing [17,18] asphalt mix additives [19,20,21,22,23], and their combinations [14,15,24,25].

Due to the aforementioned factors, increasing popularity of warm-mix asphalt techniques is seen, which created scientific interest in the properties and long-term durability of foamed mixtures. These properties of warm-mix asphalt mixtures, among other factors, are highly dependent on the composition, temperature, and aging history of bituminous binders [26,27,28]. Studies conducted during the construction of test sections using various warm-mix techniques [29] have shown that their utilization in many cases severely impacts the high-temperature grade of the asphalt binders extracted from the paved mixtures. Specifically, when lowered processing temperatures and bitumen foaming or liquid warm-mix additives were used, the asphalt binders exhibited decreased stiffness when compared to hot-mix asphalts. These observations lead to conclusions that introduction of warm-mix techniques may cause a decrease in the extent of asphalt binder ageing and, in turn, adversely affect the high-temperature performance of these asphalt mixtures in the short term [30]. This report also shows, based on data from eight different projects where a total of 12 distinct WMA techniques were utilized, that these differences in high-temperature performance are the greatest at the time of construction, and their magnitude decreases significantly in the first one to two years of pavement service. These findings were confirmed in other studies [31,32]. Because short-term ageing of asphalt binders is mainly caused by oxidation reactions between the atmospheric oxygen and the components of asphalt binder, the intensity of which is highly dependent on temperature [33], the early-life performance of warm-mix asphalts is significantly influenced by the magnitude of the decrease in production temperature. These observations are in line with the long-term performance of WMA mixtures in terms of their moisture resistance. It demonstrated that the amount of ageing may have significant effects on the moisture resistance of warm-mix asphalts, proving that adequate laboratory ageing [34] or summer ageing before the winter period [35] may be sufficient to ensure adequate performance in this scope.

In laboratory studies [26,36,37], the effects of lowered short-term ageing temperatures were found to have major impact on the high-temperature properties of all types of asphalt binders. It is also known that the ageing of asphalt binders may be influenced by factors other than those mentioned above. The use of certain additives (e.g., anti-stripping agents and liquid warm-mix additives) may slow down the aging process, limiting the increase in asphalt binder stiffness resulting from technological and long-term ageing. These phenomena were found to have source in the antioxidative and dispersive action of these additives [17,33,38]. On the other hand, solid warm-mix additives based on waxes, thanks to their distinct properties [39,40], may enhance high-temperature performance and aggregate binder adhesion in warm mixtures [41]. There is also some proof that synthetic waxes have capacity to slow down the formation of carbonyl compounds in asphalt binders and therefore decrease their ageing index [42,43]. In recent years, a number of studies [26,44,45,46,47,48] have been published regarding the effects of foaming on the properties of bituminous binders. In these works, no clear evidence was found for detrimental effects of foaming on high-temperature rheological and functional properties of bituminous binders. This tends to show that foaming alone is not responsible for the early performance of WMA mixtures.

Modern asphalt binder testing procedures, developed during the extensive research carried out during the SHRP program [49] and in later years [50,51,52], yield new parameters that were shown to be in direct relationships with many properties of the resulting asphalt mixtures. It is now known [53,54,55] that certain high-temperature properties (G*/sin(δ) and the MSCR characteristics—particularly in case of polymer modified bitumen) of the asphalt binder translate well into the high-temperature properties of the asphalt mixtures.

Based on the presented state-of-the-art in the subject area, a study was conducted to investigate the effects of three asphalt binder additives and decreased short-term ageing temperatures on two bituminous binders widely used for producing asphalt concrete courses. The paper focuses on the high-temperature properties of the investigated materials. 

## 2. Materials and Methods

### 2.1. Materials

The investigation utilized two road-paving bitumen classified, in accordance with the EN 12591 standard, as 35/50 and 50/70 binders used widely for road bases, binding courses (35/50), and wearing courses (50/70) of lightly to medium trafficked roads. The investigated binders were obtained commercially from a local refinery (Orlen Asfalt, Płock, Poland). The basic characterization of these materials is given in Table 1.

The study utilized three additives used typically in asphalt mixture technology: Wetfix BE (Nouryon, prev. AkzoNobel)—liquid anti-stripping agent and coating aid providing active and passive adhesion in hot, warm, and cold mixes [56]; Rediset LQ (Nouryon, prev. AkzoNobel)—liquid warm-mix additive permitting reductions in mixing and paving temperatures and anti-stripping effects [57]; and Sasobit (Sasol Chemicals)—a solid warm-mix additive permitting reductions in mixing and paving temperatures [58]. The main practical differences between the investigated liquid (Wetfix BE and Rediset LQ) and solid (Sasobit) additives lies in the fact that these liquid additives are said not to influence the rheological properties of the base binder when added in the recommended doing ranges, and their addition induces alteration in surface tension and modification of the interactions between the bitumen molecules based on electrochemical principles, whereas Sasobit is known to increase the stiffness of the asphalt binder at service temperatures by the physically interacting in its elastic state with the binder matrix. The rates at which the additives were added to the bituminous binders were established based on the manufacturer’s guidelines [57] as well as authors’ previous research [26].

**Figure 1:**
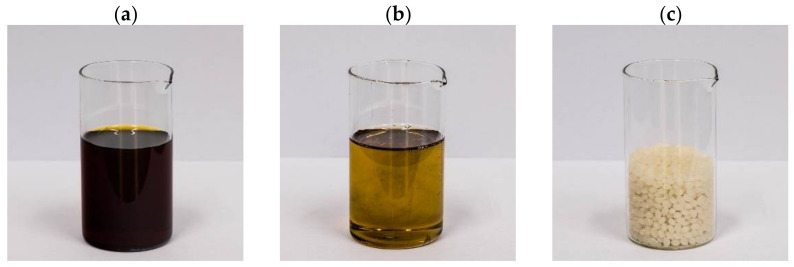
Photographs of the studied additives: (**a**) Wetfix BE, (**b**) Rediset LQ, and (**c**) Sasobit.

The additives are presented in Figure 1, and their properties are shown in Table 2.

### 2.2. Methods

#### 2.2.1. Preparation of the Asphalt Binders

The metal cans containing the asphalt binders were first heated in an oven for three hours at 145 °C and then poured into preheated, 1-dm^3^ glass containers up to approx. 500 g (with accurate mass noted). Then, the additives were added to the hot asphalt binder and mixed using high-shear homogenizer for 3 min. The binders were then kept sealed for one more hour in the oven at 145 °C, stirred gently with a glass rod, and testing samples were poured. The asphalt binders not intended for modification were heated and kept alongside the modified asphalt binders.

#### 2.2.2. Short-Term Ageing

Short-term aging of the investigated bituminous binders was carried out in a RTFOT apparatus in using a procedure based on the AASHTO T240 standard. The bituminous binders were poured into pre-heated glass containers up to a weight of 35 ± 0.5 g, and after rolling to coat the container walls, they were left to cool at ambient temperature. The RTFOT apparatus was pre-heated to test temperature (123 °C, 133 °C, 143 °C, 153 °C, 163 °C) for 2 h, after which it was filled with the containers. The time in which the test chamber reached again the testing temperature was measured, and given that it had been less than 10 min, the test was continued for another 75 min. The containers were subsequently poured, and samples for further testing were prepared. All binders were subjected to RTFOT ageing at 163 °C, and the lowered ageing temperatures were used in evaluation of only selected binders.

#### 2.2.3. Conventional Testing of Bituminous Binders

The tests for evaluating penetration at 25 °C (EN 1426) and softening point (EN 1427) were carried out in line with the respective standards using automatic apparatuses. Penetration and softening point tests were conducted with 10 and 4 replicates, respectively.

Dynamic viscosity (EN 13302) was measured using CAP2000 Brookfield rotary viscometer model DV-II+PRO with SC4-27 spindle in temperature increments of 10 °C, with the lowest temperature being 90 °C, the highest 150 °C, and an additional measurement at 135 °C according to AASHTO MP-1. Dynamic viscosity testing was conducted with 2 replicates.

The tests for evaluating conventional properties of the bituminous binders were conducted on non-aged binders. 

#### 2.2.4. High-Temperature Functional Properties of Bituminous Binders

The evaluation of high-temperature functional characteristics of the investigated binders was performed using Rheotest RN4 (Rheotest Medingen GmbH, Ottendorf-Okrilla, Germany) dynamic shear rheometer. Two types of tests were used to broadly characterize the binders: oscillatory tests were used for evaluating binder stiffness in the linear-viscoelastic domain, and multiple stress creep recovery tests investigated their creep characteristics. Binder samples were prepared with the guidance of the AASHTO T315 standard using silicone-rubber molds.

The oscillatory tests for measuring complex stiffness modulus G* were conducted in line with the AASHTO T315 standard, and the testing was carried out from the lowest to the highest temperature, with a 6 °C step (from 52 °C to 82 °C). Testing was performed in controlled stress mode, with stress levels selected in a way that the linear-viscoelastic response of the binders was obtained. The loading frequency was 1.59 Hz (approx. angular frequency of 10 rad/s). The data were also used to calculate the high critical temperature, that is, the temperature at which the binders exhibited G*/sin(δ) = 2.2 kPa after short-term ageing under the RTFOT protocol only. 

The multiple stress creep recovery tests were carried out in line with the AASHTO T350 standard, and non-recoverable creep compliance was calculated based on the results of the loading series. The tests were performed at 58 °C and 64 °C relating to the high pavement temperatures. The non-recoverable creep compliance measured at a 3.2 kPa stress level (J_nr 3.2 kPa_) was selected for the evaluation of the binders based on the high correlation of this parameter with rutting performance of asphalt mixtures [53,60]. Recovery was not evaluated in detail due to the nature of the investigated binders (lacking elastomeric modification). All dynamic shear rheometer testing was conducted with 4 replicates.

#### 2.2.5. Statistical Analysis of the Results

The results were analyzed using the Statistica (TIBCO Software Inc.) software package. The analysis involved analysis of variance, evaluating normality of the residuals, evaluation of variance, and HSD Tukey tests to discriminate homogenous groups within the tested binders. In all cases, the prerequisites for specific tests were met, and only the results of the HSD Tukey tests are shown. The presented figures show mean measured values and their 95% confidence intervals (whiskers) of the investigated properties.

## 3. Results

The following subsections provide the results of the conducted tests. A specific notation is used to characterize the investigated binders according to the scheme XXYY-Z, where:

- XX—marks the type of the bitumen by its penetration range:
  ◯ 35—35/50 penetration bitumen,
  ◯ 57—50/70 penetration bitumen;
- YY—marks the type of the additive used:
  ◯ AS—anti-stripping agent (Wetfix BE) [56],
  ◯ WL—liquid WMA additive (Rediset LQ) [57],
  WS—solid WMA additive (Sasobit) [58];
- Z—marks the amount of the additive used:
  ◯ L—low value (0.3% of AS and WL, 1.5% of WS),
  ◯ H—high value (0.6% of AS and WL, 3.0% of WS).

### 3.1. Conventional Propetries of the Bituminous Binders

**Figure 2:**
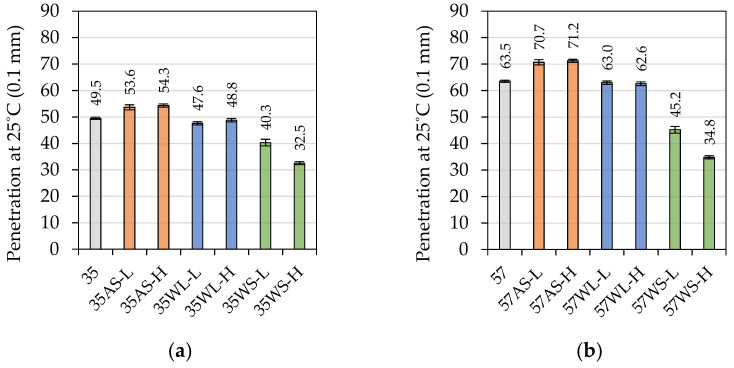
Penetration at 25 °C of the investigated asphalt binders based on the (**a**) 35/50- and (**b**) 50/70-penetration bitumen.

The results of the penetration tests at 25 °C are presented in Figure 2, and their statistical analysis is presented in Table 3.

The tested reference 35/50 and 50/70 penetration binders were characterized by penetrations of 49.5 × 0.1 mm and 63.5 × 0.1 mm, respectively. The presented results and the results of statistical analysis show that the influence of the tested additives on this parameter depended on the type of the modifier. In the case of both base binders, the anti-stripping agent (AS) caused a significant increase of their penetration values, which was similar at both low and high level of dosing. On the other hand, the application of the liquid WMA additive (WL) had only a little effect on the measured values of the binders’ penetration. This was especially visible in the case of the 50/70 bitumen, where the 57, 57WL-L, and 57WL-H binders were assigned, in the HSD Tukey statistical test, to a single homogenous group. The solid WMA additive (WS, Sasobit) had very distinct effect on this characteristic of both evaluated binders, causing a decrease of penetration values nearly proportional to the amount of the additive. This resulted in the penetration to be measured at 32.5 × 0.1 mm and 34.8 × 0.1 mm for the 35WL-L and 57WL-H binders, respectively.

**Figure 3:**
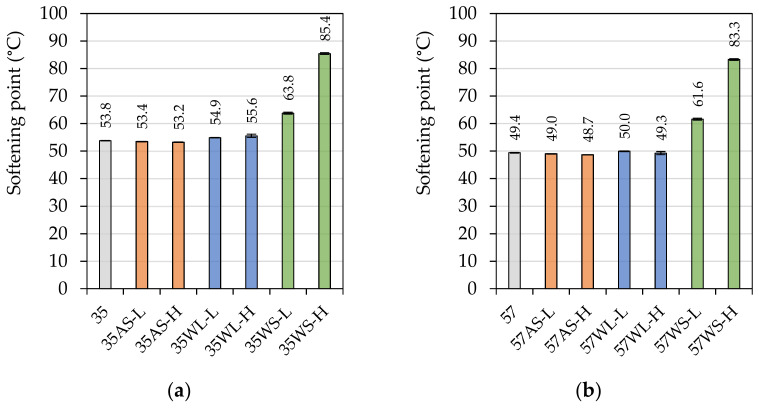
Softening point of the investigated asphalt binders based on the (**a**) 35/50- and (**b**) 50/70-penetration bitumen.

Figure 3 presents the results of the investigated binders’ softening point measured using the ring and ball method, and the results of the statistical analysis are presented in Table 4.

In this scope, the liquid additives showed to have less effect on the tested bitumens than it was seen in case of penetration. The anti-stripping agent had no statistical effect (Table 4) on the values of softening point in the 35/50 binders, while the liquid WMA additive (WL) only slightly contributed to the increase of this characteristic. In the case of the 50/70 binders, the liquid additives had no effect on this characteristic. On the contrary, the addition of the solid WMA additive resulted in very significant increases of softening point in both binders, with the effect being stronger when its concentration increased from 1.5% to 3%.

**Figure 4:**
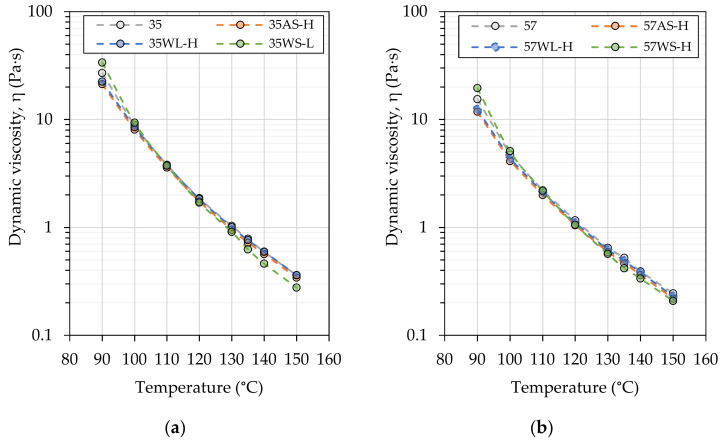
Dynamic viscosity of the investigated asphalt binders based on the (**a**) 35/50- and (**b**) 50/70-penetration bitumen.

Figure 4 presents the results of viscosity measurements performed on selected bituminous binders, which utilized the high (-H) dosing of liquid additives and different dosing of the solid WMA additive—low (1.5%) with the harder, 35/50 bitumen and high (3.0%) with the softer, 50/70-penetration binder.

The determination of dynamic viscosity showed that in the case of both base bitumens, the liquid additives resulted in slight decreases of this parameter throughout the evaluated temperature range, while the solid WMA additive caused significant, complex changes in their viscosity. At temperatures above the 100–110 °C, WS-modified binders exhibited higher viscosities than base binders, while at higher temperatures, their dynamic viscosities were visibly decreased. Table 5 shows the estimated processing temperatures of asphalt mixtures based on the results of dynamic viscosity measurements and the requirements of [61] based on the principle of equiviscosity. The results show that the use of the investigated binders enable decreases in the production and paving temperatures; however, in the case of WL and WS additive, they are significantly smaller than those reported by the manufacturers of these additives.

### 3.2. High-Temperature Functional Properties of Bituminous Binders

**Figure 5:**
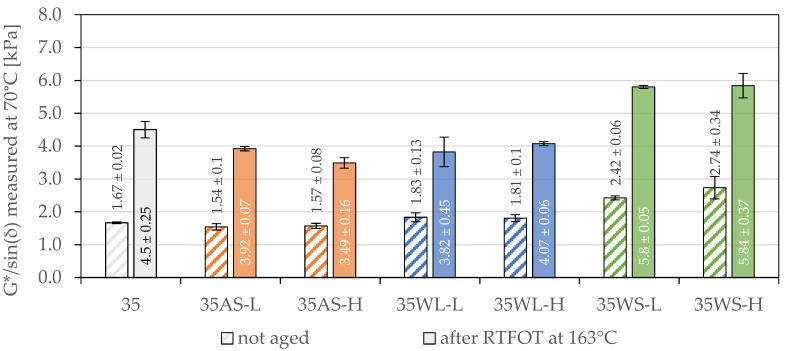
High-temperature stiffness of the investigated asphalt binders based on the 35/50-penetration bitumen.

**Figure 6:**
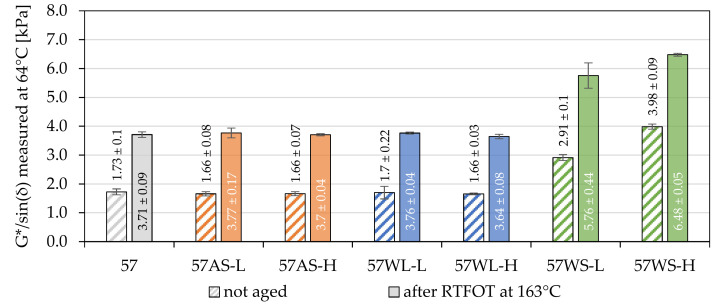
High-temperature stiffness of the investigated asphalt binders based on the 50/70 penetration bitumen.

The results of the high-temperature stiffness (G*/sin(δ)) of the investigated binders based on the 35.50 and 50/70 paving-grade bitumens are presented in Figure 5 and Figure 6. The measured values were obtained at temperatures equal to the high-temperature grades (PG) of the base binders. The results of the statistical analyses are presented in Table 6.

In the case of bituminous binders based on the 35/50 paving-grade bitumen, the use of liquid additives resulted in significant changes in their high-temperature stiffness. Moreover, these changes were dependent on the short-term RTFOT aging. The most significant effects were observed when the anti-stripping agent was used, which did not cause a significant change in the stiffness of the unaged binder; however, the G*/sin(δ) values determined after RTFOT aging were significantly lower than in the case of the base bitumen. These changes were also negatively correlated with the concentration of the AS additive. For comparison, the use of liquid additives in the composition of 50/70 road-paving bitumen had no effect on its high-temperature stiffness both before and after RTFOT aging. 

The effect of using the WS additive on the high-temperature stiffness of the tested asphalt binders was statistically significant, and it varied depending on the base bitumen. The difference between the values of the G*/sin(δ) parameter of the 35WS-L and 35WS-H binders after RTFOT was so small that its statistical significance was not proven. At the same time, significant differences were noted in the stiffness of these binders before short-term aging. In the case of the base 50/70 paving-grade bitumen, the effect of increasing the amount of WS additive to 1.5% and 3.0% was significant regardless of the short-term aging.

To quantify the effects shown in Figure 5 and Figure 6, the comparison of the relative changes in the G*/sin(δ) parameter values are shown before and after short-term aging caused by the use of the analyzed additives in Table 7. In the 35/50 base asphalt binder, both the AS and the WL liquid additive limited the aging processes caused by RTFOT ageing. Such a statement is possible thanks to the observation of the significantly reduced high-temperature stiffness of the 35AS and 35WL binders after short-term aging in relation to the 35/50 and 50/70 binders without these additives. This effect was not observed for the 50/70 asphalt binder. 

Regarding the effects of the WS additive, it was observed that the stiffening of both asphalt binders due to its addition measured after short-term RTFOT aging was significantly lower than before aging. This difference ranged from 16.6 to 34.6 percent points for 35/50 asphalt binders and from 13.5 to 54.2 percent points for the 50/70 asphalt binders. These results indicate that the addition of synthetic wax had a significant impact on the course of short-term aging processes for both binders. It can also be concluded that the evaluation of the effect of the application of this additive on the basis of tests of non-aged binders may lead to erroneous overestimation of the stiffening of the binder caused by this additive. The conclusions above, in particular the differences in changes in the G*/sin(δ) values of binders before and after short-term aging, presented in Table 7, indicate that the additives significantly affected the binders’ high-temperature stiffness in scope of the short-term RTFOT aging.

**Figure 7:**
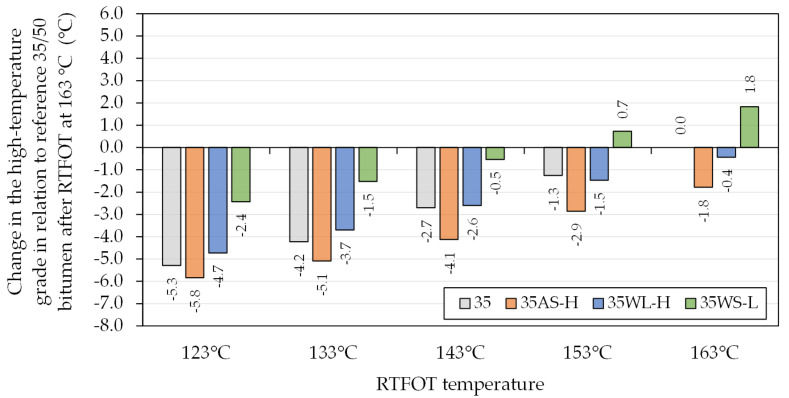
Change in the high-temperature grade of the investigated asphalt binders based on the 35/50-penetration bitumen due to RTFOT ageing in relation to the reference 35/50 bitumen after RTFOT at 163 °C.

**Figure 8:**
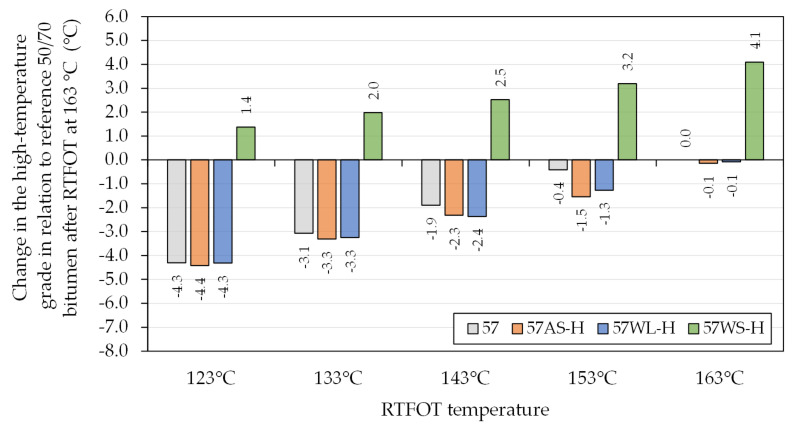
Change in the high-temperature grade of the investigated asphalt binders based on the 50/70-penetration bitumen due to RTFOT ageing in relation to the reference 50/70 bitumen after RTFOT at 163 °C.

The oscillatory measurements taken using the dynamic shear rheometer were also used to determine the high critical temperatures of the base asphalt binders (Table 1). The selected binders were evaluated for the effects of the additives in conjunction with the decreased short-term aging temperatures on this parameter. Figure 7 and Figure 8 present these effects in relation to the reference binders aged at the typical temperature of 163 °C. 

The combined use of decreased ageing temperatures and different additives had significant effects on the stiffness and thereby the high critical temperature (continuous high PG grade) of the investigated asphalt binders. The lowered ageing temperatures resulted in major changes of the high critical temperature of the investigated binders (G*/sin(δ) = 2.2 kPa), which were nearly proportional to the lowering of RTFOT temperatures. In many cases, the change of the continuous grades of the asphalt binders was greater than −3 °C (half of a grade), which potentially may lead to a change in the classification of the bitumen. These effects, when the WS additive was used, were mostly negated by its characteristics, which increased the stiffness of the bitumen. On the other hand, the use of AS and WL had minor negative effects on the high critical temperatures of the asphalt binders.

**Figure 9:**
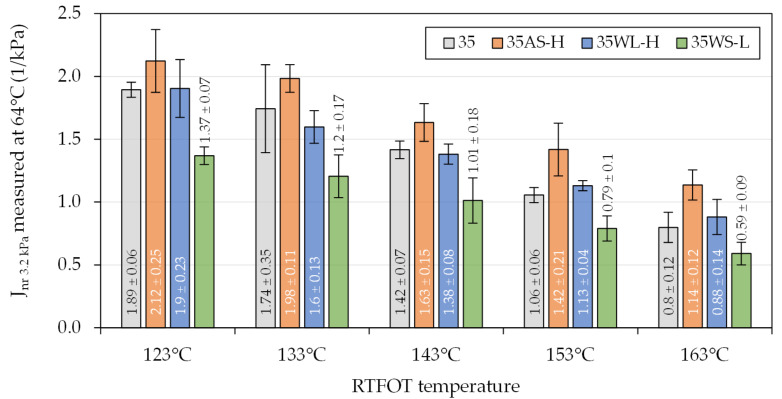
Non-recoverable creep compliance of the investigated asphalt binders based on the 35/50-penetration bitumen evaluated in the MSCR test.

**Figure 10:**
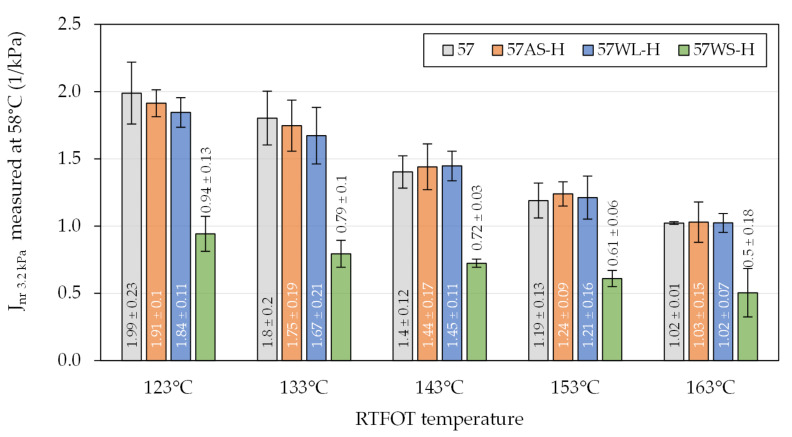
Non-recoverable creep compliance of the investigated asphalt binders based on the 50/70-penetration bitumen evaluated in the MSCR test.

Similar evaluation was conducted in regard to the multiple stress creep recovery (MSCR) characteristic—non-recoverable creep compliance measured at a 3.2 kPa stress level (J_nr 3.2 kPa_)—of the selected binders (Figure 9 and Figure 10). The presented figures show the test results of the 35/50 and 50/70 bitumen-based binders obtained at temperatures of 64 °C and 58 °C, respectively. The temperatures were selected to obtain comparable results despite the differences in the stiffness of the binders.

When the binders were exposed to a standard RTFOT ageing procedure with the temperature of 163 °C, the resulting J_nr_ values were very low (typically ≤ 1 × 1/kPa), but when the ageing temperatures were decreased to 123 °C, the non-recoverable creep compliance increased nearly twofold, up to approx. 2 × 1/kPa. Following the results of oscillatory testing, the 35/50 bitumen-based binders were more strongly affected by the liquid additives, in particular the anti-stripping agent (AS), which caused significant increases in the non-recoverable compliance. Smaller effects of those additives were observed in the 50/70-based binders. The solid WMA additive added to both of the base binders caused major decreases in the J_nr_ values, which in the case of the 50/70 binder, amounted to approx. 50%. In effect, asphalt binders with the WS additive were by overall less affected by the decrease in the RTFOT ageing temperatures. 

**Figure 11:**
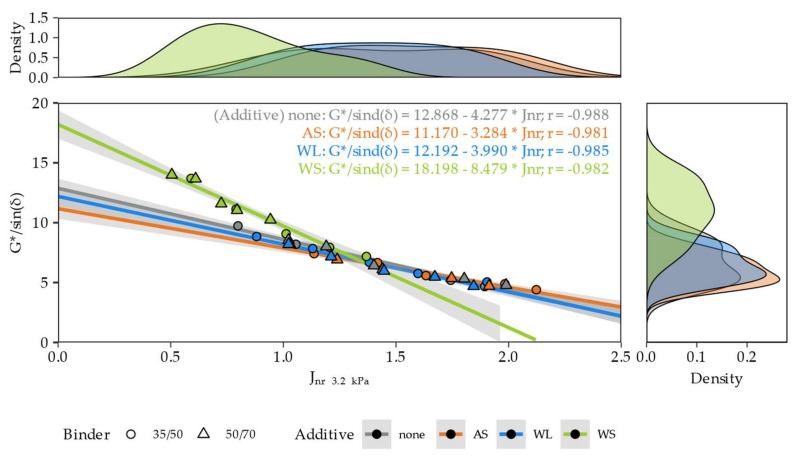
Correlations between the high-temperature stiffness (G*/sin(δ)) and non-recoverable creep compliance (J_nr 3.2 kPa_) measured at 64 °C for the 35/50 bitumen-based binders and at 58 °C for the 50/70 bitumen-based binders.

The visibly different characteristics of the asphalt binders with the WS additive were observed also when the high-temperature stiffness (G*/sin(δ)) and non-recoverable creep compliance J_nr 3.2 kPa_ were compared. The correlations between these two characteristics are shown in Figure 11.

The relationships between the G*/sin(δ) and J_nr 3.2 kPa_ parameters were highly linear, and their correlations were strongly negative (Pearson correlation coefficients *r* were between −0.981 and −0.988). These relationships were similar in the case of neat binders and binders with liquid additives (AS, WL). This characteristic of the asphalt binders with the WS additive was distinctly steeper and, as seen on the density plots, shifted towards lower non-recoverable creep compliances and higher values of the high-temperature stiffnesses. The type of the base binder (35/50, 50/70) was found to poorly discriminate the G*/sin(δ) vs. J_nr 3.2 kPa_ characteristics. 

## 4. Discussion

The utilization of lowered production and paving temperatures in warm-mix asphalts brings many economic and ecological benefits, as shown in the introduction. However, successful application of these techniques requires additional considerations. The utilization of warm-mix asphalt techniques may lead in some cases to a decrease in high-temperature performance of these mixtures not due to their perceived lower quality but due to insufficient ageing of the asphalt binder. 

Ageing processes, often regarded only as a detrimental factor in the production processes, play a key role in the asphalt-mix technology. The stiffening effect that short-term (technological) ageing has on the asphalt binder is indispensable for the final mix to be able to withstand heavy traffic without exhibiting excessive permanent deformations. 

Lowered temperatures during the production of warm-mix asphalt impede the action of oxygen by, among other mechanisms, limiting the oxidation reaction rates and slowing the permeation of oxygen into the bitumen which exhibits at lower temperature and higher viscosity [33]. These effects, when not accounted for, in some cases may lead to reliability issues in the early stages of pavement life.

The results obtained in this study should be analyzed in view of the results obtained during actual construction of pavements using warm-mix techniques. Such results were presented by West et al. [29], showing effects of WMA processes on the high-temperature grade of asphalt binder across different test sections and WMA technologies. It was found that the mean change of asphalt binder high critical temperature, compared to HMA, amounted to −2.3 °C, while the single greatest decrease was recorded at −5.7 °C. The greatest decreases in the bitumen high-temperature grades was observed when liquid WMA additives and bitumen foaming were used. These results, viewed in obviously narrow scope of the present study, show that these effects correspond to laboratory RTFOT short-term ageing carried out at 133–143 °C.

As shown in this study, asphalt binders and mixtures for WMA techniques should be scrutinized for their functional performance under specific short-term ageing conditions. The presented results indicate that the susceptibility of the asphalt binders to decreased processing temperatures and WMA processes cannot be reliably predicted in conventional testing. This issue is even more important given that asphalt binders from different sources may exhibit major variability in their rheological properties, susceptibility to ageing. and chemical composition. It is also important to note that most studies do not take into account the effects of anti-stripping agents, which, as shown in [62] and the present study, may cause significant changes in the rheological behavior of certain asphalt binders, e.g., due to the introduction of slip surfaces between bitumen molecules [63]. All mentioned effects, as shown in a recent study [64], can be accurately evaluated, e.g., in DSR testing.

## 5. Conclusions

The present paper investigated the effects of lowered short-term ageing temperatures on the properties and high-temperature performance of different asphalt binders intended for producing warm-mix asphalts. The study, which included two paving-grade bitumen (35/50 and 50/70) and three types of additives (anti-stripping agent; liquid warm-mix additive; and solid, wax-based warm-mix modifier) highlighted that the use of additives that improve the mix’s resistance to permanent deformations, harder grades of asphalt binders, and/or more elastic asphalt binders may be necessary to ensure the reliability of asphalt courses processed with lowered temperatures. In this view, it was shown that it is possible to successfully counteract the decreased severity of binder ageing by introducing additives like Sasobit, which interacts with the bituminous binder physically by solidifying in-service temperatures and therefore increasing the stiffness of the binder.

To bring fuller understanding into this topic, comprehensive studies are needed. The future work in this field should include asphalt binder and asphalt-mix laboratory ageing as well as the analysis of the binder in the paved mixtures. In future studies, it is crucial to include different binders (paving-grade bitumens, polymer-modified bitumens) while taking into account the actual production and paving temperatures as well the effects related to the utilized additives.

## Figures and Tables

**Table 1:** Characterization of the reference 35/50 and 50/70 road-paving bitumen used as a base for the investigated binders.

Binder Type		35/50 (“35”)	50/70 (“57”)
Penetration, EN 1426, (0.1 mm)		49.5	63.5
Softening point, EN 1427, (°C)		53.8	49.4
Frass breaking point, EN 12593, (°C)		−13.0	−14.0
Dynamic viscosity, EN 13302, (Pa·s)	at 105 °C at 120 °C at 135 °C at 150 °C	6.30 1.87 0.78 0.36	3.65 1.16 0.52 0.25
High PG (continuous), ASTM D 7643, (°C)		75.4	68.5
Performance grade, AASHTO M 332, (°C)		70–22	64–22

**Table 2:** Characterization of the reference 35/50 and 50/70 road-paving bitumen used as a base for the investigated binders.

Property	Wetfix BE [56]	Rediset LQ [57,59]	Sasobit [58,59]
Form	viscous liquid	viscous liquid	solid pellets
Color	dark brown	brown	white
Density (kg/m^3^)	0.980	0.975	0.902
Viscosity at 20 °C (mPa∙s)	538	471	n/a
Dosing range (by wt. of asphalt binder)	0.1–1.0%	0.3–0.8%	1–4%
Congealing point (°C)	n/a	n/a	100–110
Penetration at 25 °C (0.1 mm)	n/a	n/a	0–2

**Table 3:** Results of Tukey HSD tests for distinguishing homogenous groups (penetration at 25 °C, α = 0.05) within the tested bituminous binders.

Tukey HSD Test, Homogenous Groups, α = 0.05. Variable: Penetration at 25 °C										
Groupings	1	2	3	4	5		1	2	3	4
35		2				57	1			
35AS-L			3			57AS-L		2		
35AS-H			3			57AS-H		2		
35WL-L	1					57WL-L	1			
35WL-H	1	2				57WL-H	1			
35WS-L				4		57WS-L				4
35WS-H					5	57WS-H			3	

**Table 4:** Results of Tukey HSD tests for distinguishing homogenous groups (softening point, α = 0.05) within the tested bituminous binders.

Tukey HSD Test, Homogenous Groups, α = 0.05. Variable: Softening Point								
Groupings	1	2	3	4		1	2	3
35	1				57	1		
35AS-L	1				57AS-L	1		
35AS-H	1				57AS-H	1		
35WL-L		2			57WL-L	1		
35WL-H		2			57WL-H	1		
35WS-L			3		57WS-L		2	
35WS-H				4	57WS-H			3

**Table 5:** Processing temperatures of asphalt mixtures estimated based on the dynamic viscosity measurements.

Asphalt Binder	35	35 AS-H	35 WL-H	35 WS-L	57	57 AS-H	57 WL-H	57 WS-H
Compaction (maximum, °C)	145	143	144	142	135	133	134	132
Mixing (minimum, °C)	158	157	158	154	154	149	150	147

**Table 6:** Results of Tukey HSD tests for distinguishing homogenous groups (G*/sin(δ), α = 0.05) within the tested bituminous binders.

Tukey HSD Test, Homogenous Groups, α = 0.05. Variable: G*/sin(δ)															
Ageing	Not Aged				RTFOT (163 °C)					Not Aged			RTFOT (163 °C)		
Groupings	1	2	3	4	1	2	3	4		1	2	3	1	2	3
35	1	2						4	57	1			1		
35AS-L	1				1				57AS-L	1			1		
35AS-H	1					2			57AS-H	1			1		
35WL-L		2			1	2			57WL-L	1			1		
35WL-H		2			1				57WL-H	1			1		
35WS-L			3				3		57WS-L		2			2	
35WS-H				4			3		57WS-H			3			3

**Table 7:** Effects of the investigated additives on the high-temperature stiffness (G*/sin(δ)) of the 35/50 and 50/70 binders before and after short-term laboratory aging.

Binder	35/50: G*/sin(δ) (70 °C)		50/70: G*/sin(δ) (64 °C)	
Additive	Not Aged	RTFOT at 163 °C	Not Aged	RTFOT at 163 °C
none	100.0%	100.0%	100.0%	100.0%
AS-L	92.6%	87.1%	96.0%	101.5%
AS-H	94.4%	77.5%	96.3%	99.9%
WL-L	110.1%	84.9%	98.4%	101.4%
WL-H	108.6%	90.5%	95.9%	98.2%
WS-L	145.4%	128.8%	168.7%	155.2%
WS-H	164.3%	129.7%	230.8%	174.6%

## Data Availability

Data available on request.
